# The concentration and origins of carboxylic acid groups in oil paint†

**DOI:** 10.1039/c9ra06776k

**Published:** 2019-11-01

**Authors:** Lambert Baij, Louise Chassouant, Joen J. Hermans, Katrien Keune, Piet D. Iedema

**Affiliations:** Van 't Hoff Institute for Molecular Sciences, University of Amsterdam PO box 94720 1090GD Amsterdam The Netherlands C.L.M.Baij@uva.nl +31 20 525 7143; Rijksmuseum Amsterdam, Conservation and Science PO box 74888 1070DN Amsterdam the Netherlands

## Abstract

Although the concentration of carboxylic acid (COOH) groups is crucial to understand oil paint chemistry, analytical challenges hindered COOH quantification in complex polymerised oil samples thus far. The concentration of COOH groups is important in understanding oil paint degradation because it drives the breakdown of reactive inorganic pigments to dissolve in the oil network and form metal carboxylates. The metal ions in such an ionomeric polymer network can exchange with saturated fatty acids to form crystalline metal soaps (metal complexes of saturated fatty acids), leading to serious problems in many paintings worldwide. We developed two methods based on ATR-FTIR spectroscopy to accurately estimate the COOH concentration in artificially aged oil paint models. Using tailored model systems composed of linseed oil, ZnO and inert filler pigments, these dried oil paints were found to contain one COOH group per triacylglycerol unit. Model systems based on a mixture of long chain alcohols showed that the calculated COOH concentration originates from side chain autoxidation at low relative humidity (RH). The influence of increasing RH and ZnO concentration on COOH formation was studied and high relative humidity conditions were shown to promote the formation of COOH groups. No significant ester hydrolysis was found under the conditions studied. Our results show the potential of quantitative analysis of oil paint model systems for aiding careful (re)evaluation of conservation strategies.

## Abstract 2

Zinc complexation can be used to quantify the concentration of carboxylic acid groups in an oil painting.

## Graphical abstract

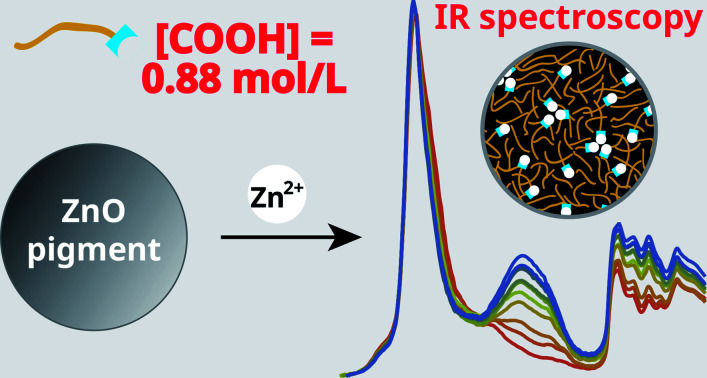

## Introduction

The degradation of oil paintings manifests itself by visible and mechanical paint alterations such as brittleness, loss of opacity, formation of protrusions, and delamination of paint layers.^1^ These alarming phenomena have initiated the chemical analyses of many paint samples over the past decades. However, thorough understanding of the underlying chemical and physical mechanisms causing paint degradation started only recently.^2–6^ The chemical composition of a mature oil paint binder has proved to play an essential role in several of these degradation phenomena. Understanding the driving forces behind degradation processes will ultimately provide useful knowledge for the preservation and conservation of oil paintings.

We have shown previously that in a mature oil paint binding medium, carboxylic acid (COOH) groups often bind to metal ions (originating from pigments or driers) and form an ionomeric polymer network.^2,3^ The ionomeric binding medium, like commercial ionomers,^7^ contains clusters of metal carboxylates (COOM), often identified by a broad asymmetric *ν*_a_ COO^−^ infrared (IR) absorption band.^3,6^ These ionomeric metal carboxylate complexes were discovered to represent an intermediate stage in paint ageing that can ultimately lead to the appearance of crystalline metal soaps (metal complexes of long chain saturated fatty acids).^5^ Metal soaps play an important role in many types of oil paint degradation.^1^

The concentration of COOH groups in polymerised oil is expected to be a crucial factor affecting the extent of oil paint degradation. Being an integral part of the mature oil paint binding medium, COOH groups are most likely the driving force for the release of metal ions by inorganic pigments and the formation of (network-bound) COOM.^3^ Furthermore, COOH groups indirectly determine the extent of metal soap formation, because before metal soap crystallisation can occur, network-bound COOM need to exchange with free SFAs.^5^ Although many^8–16^ have studied *extractable* acids, the concentration of COOH groups linked to the polymer network has, to the best of our knowledge, never been quantified.

**Fig. 1:**
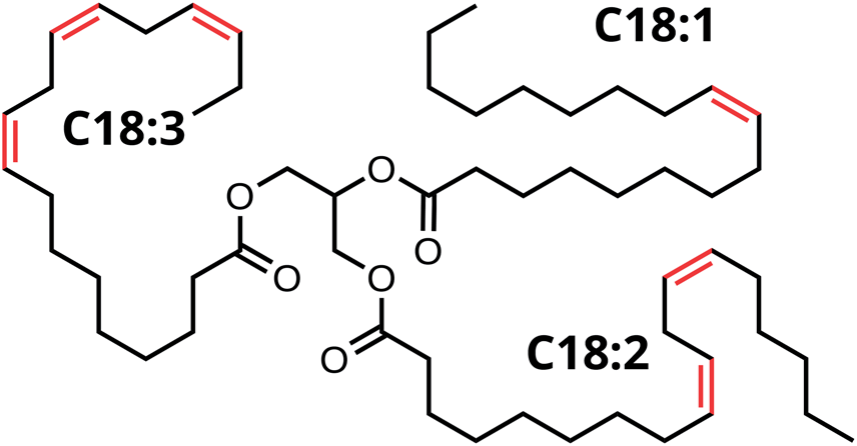
Structure of a triacylglyceride (TAG) unit in linseed oil (LO), C18:3 denotes linolenic acid, C18:2 linoleic acid and C18:1 oleic acid.

**Scheme 1:**
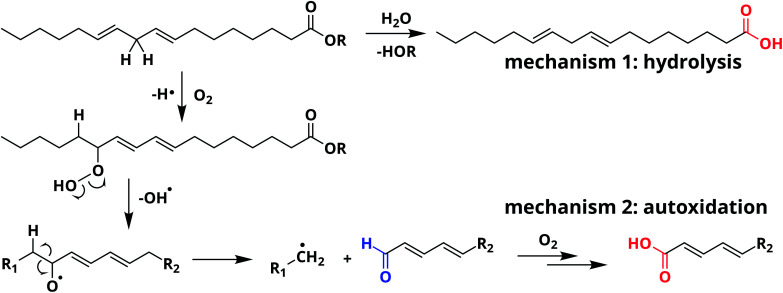
Two mechanisms that lead to the formation of acid groups (in red) starting from the ester of linoleic acid: (1) hydrolysis and (2) autoxidation of side chain C=C bonds. Note that autoxidation leads to additional products, including aldehydes (in blue).

From a chemical point of view, oil paint is a mixture of mainly inorganic pigments, a drying oil consisting of triacylglycerides (TAGs) and a variety of possible additives. Linseed oil (LO) is widely used in oil paintings because it possesses excellent drying properties. LO consists of a mixture of TAGs, mostly containing linolenic acid (C18:3), linoleic acid (C18:2), and oleic acid (C18:1) side chains (see Fig. 1). As the oil undergoes autoxidation reactions,^17–19^ the paint mixture becomes a complex heterogeneous polymer with solid pigment particles suspended in the densely cross-linked network. This polymer network contains mainly ether- or peroxy-type cross-links formed through autoxidation of double bonds on fatty acid side chains. Over time, higher oxidation products of these oxygen cross-links result in the formation of aldehydes and COOH groups (see Scheme 1). It is important to note that COOH groups are a product of the autoxidation process (mechanism 2). Consequently, the ionomeric state is also found in relatively young oil paints. Upon ageing, hydrolysis of ester bonds can introduce additional COOH groups (mechanism 1). Since the two different mechanisms of COOH formation may dominate at different stages during the lifetime of an oil paint, we aim to distinguish them in our experiments.

The present paper focuses on the crucial role COOH groups play in pigment degradation. Quantifying the amount of COOH groups attached to a heterogeneous polymer network is a challenging task. Most spectroscopic, electrochemical or calorimetric methods to determine the acid concentration in vegetable oils require a homogeneous solution of the (non-polymerised) oil in a solvent and only work for free fatty acids (FFAs).^20,21^ Because we are interested in the COOH groups linked to the polymer network, the conventional methods of breaking up TAGs using pyrolysis GC/MS do not give the desired information.^22^ Potentiometric titration of COOH groups^23^ proved to be time consuming and inaccurate since polymerised LO is extremely insoluble and the diffusion of water is very slow.^24^ Methods based on derivatization with fluoride transfer reagents^25,26^ showed the existence of acid groups but did not give a reproducible conversion when applied to our polymeric materials. Swollen-state ^13^C NMR spectroscopy also enabled the detection of acid groups but was not used in a quantitative manner.^27^

Attenuated total reflection Fourier-transform infrared (ATR-FTIR) spectroscopy is a powerful tool for the study of organic polymers and solid paint materials. However, ATR-FTIR does not allow for a direct observation of the COOH concentration in polymerised LO due to the overlapping IR absorption bands of the asymmetric carbonyl stretching vibration of the ester (COOR, 1738 cm^−1^), aldehyde (COH, 1726 cm^−1^) and carboxylic acid (COOH, 1710 cm^−1^) groups (see Fig. S1†). Simple peak deconvolution is unreliable because additional overlapping absorption contributions from FFAs, ketones and *ν*(C=C) bonds are present in this spectral region.^28^ A practical solution is the complexation of COOH groups with Zn^2+^ ions (released by ZnO), leading to the appearance of a broad, free-lying IR absorption band for zinc carboxylates (COOZn) centered around 1585 cm^−1^.^2,6^ We have recently unequivocally assigned the structure and coordination in these COOZn complexes: they adopt either a coordination chain- or an oxo-type cluster structure.^6^ The COOZn IR absorption band can be used as a method to accurately estimate the concentration of COOH in polymerised LO that is accessible for reaction with zinc ions. This concentration is most relevant for oil paint degradation since amorphous COOZn is the intermediate in crystalline metal soap formation. Throughout this text, the COOZn band will be used as a measure for COOH concentration.

In our experiments, we monitor COOZn formation by varying the amounts of ZnO or COOH in a controlled manner. In one series of experiments, the ZnO concentration is increased at constant COOH concentration. In a second series of experiments, the acidity is varied under excess ZnO. Because acid groups in polymerised LO can form according to two mechanisms: (1) hydrolysis of ester bonds and (2) autoxidation of side chains double bonds (see Scheme 1), we also investigate which mechanism of COOH group formation dominates. Regular LO does not allow to discriminate between these two mechanisms of acid formation. We employ a mixture of alcohols obtained by reducing LO with LiAlH_4_, blocking the hydrolysis pathway and forming acid groups by autoxidation only. Because the concentration of acid groups in our model systems is not universally valid for aged paintings, we investigate how environmental conditions affect the COOH concentration. More specifically, the influence of ZnO on ester hydrolysis is investigated by examining samples at low and high relative humidity (RH) as a function of ZnO concentration.

In this paper, we develop an analytical method to quantify the concentration of COOH groups attached to a complex polymerised oil network. Knowing the quantity and origins of COOH groups can lead to improved storage and conservation strategies and an extended lifetime of invaluable works of art.

## Materials and methods

### Sample preparation

Model paint samples containing ZnO (Sigma Aldrich, ≥99%) and coated rutile TiO_2_ (Sigma Aldrich, >99.9%) or ZnO and BaSO_4_ (Kremer pigmente 58700, ≥98%) were made by grinding the pigments with cold-pressed untreated linseed oil (Kremer pigmente) in a 1 : 1 (w/w) ratio to a smooth paste with mortar and pestle. SEM images of pigment particles are shown in Fig. S4.† The successful reduction of the esters in LO to alcohols was achieved using LiAlH_4_ in THF (see Fig. S7 and ESI† for details). Care was taken to keep the Pigment Volume Concentration (PVC) at 17–19% in all series. The mixture was applied to 50 × 75 mm glass slides and spread with a draw-down bar to achieve a wet thickness of 190 μm. The samples were cured in the dark in air at 60 °C for 7 days at different RH. RH was controlled using a saturated NaCl solution (for 77% RH) in a closed container and was determined using a Rotronic HL-1D temperature and humidity data logger. For all measurements, 5 × 5 mm squares of the films were cut and lifted off the glass support for ATR-FTIR analysis. The exact sample composition is given in Tables S1–S6.†

### ATR-FTIR spectroscopy

ATR-FTIR spectra were measured on a PerkinElmer Frontier FT-IR spectrometer fitted with a Pike GladiATR module equipped with a heated top plate and a diamond ATR-crystal (*ø* = 3 mm). Spectra were averaged over 4 scans. To integrate overlapping absorption bands, automated data correction and integration algorithms were written using Wolfram Mathematica software. For integration, IR spectra were averaged over either 3 or 5 measurements and the baseline was set to zero at 1820 cm^−1^. Spectra were normalised on the ester carbonyl (1740 cm^−1^) or CH_2_ (2920 cm^−1^) band, after which the ester CO band was subtracted and integration performed between 1500–1650 cm^−1^. An estimated non-linear baseline correction was used to remove TiO_2_ absorption. An illustration of the background subtraction and band fitting procedures is given in Fig. S2 and S3.† In all graphs, error bars due to spectral variation are smaller than the symbols.

### X-ray diffraction

X-ray diffraction (XRD) measurements on cured films were recorded with a Rigaku MiniFlex II desktop X-ray diffractometer using Cu Kα radiation at 2.5° min^−1^ on *ca.* 10 × 10 mm squares of paint film taped to a glass sample holder.

## Results and discussion

### COOH concentration in ZnO paints

#### Paints with increasing ZnO content

To explore the effect of ZnO concentration on COOZn formation, mixed pigment paint models containing various amounts of ZnO and a BaSO_4_ filler were prepared (denoted LO–ZnO–BaSO_4_). The BaSO_4_ pigment is a well-known inert filler^29^ for white paints with a comparable density to ZnO (ZnO and BaSO_4_: 5.6 and 4.5 g cm^−3^, respectively). Within the series, the total PVC was kept constant. The exact sample composition is given in Table S1.†

**Fig. 2:**
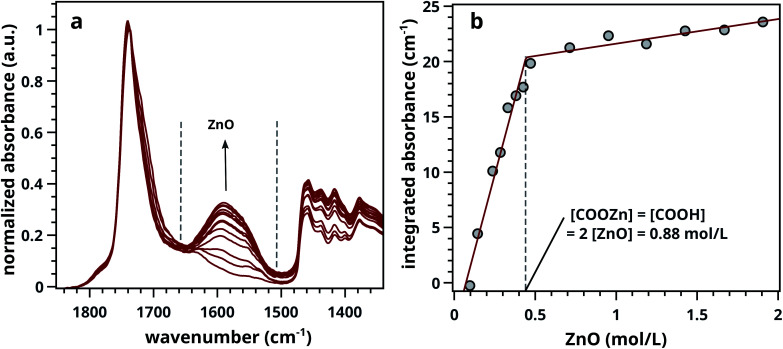
(a) Normalised ATR-FTIR spectra for LO–ZnO–BaSO_4_ from 0.14–1.8 mol L^−1^ of added SA (0.0–20 wt%). Dashed lines indicate band integration limits. (b) Integrated absorption values of COOZn band, clearly showing the ‘tipping point’ (dashed line) at 0.44 mol L^−1^ ZnO marking complete COOH neutralisation. Further increase of ZnO does not increase the amount of COOZn. See Fig. S2† for details on spectral processing.

Ester normalised FTIR spectra for samples containing 0.5–20 wt% of ZnO are depicted in Fig. 2a, showing an increasing concentration of COOZn with increasing ZnO content. Integrated absorption values for the *ν*_a_ COOZn IR absorption band at 1585 cm^−1^ are plotted as a function of ZnO concentration in Fig. 2b. Two regimes are visible in Fig. 2b, separated by a clear ‘tipping point’: a steep increase in COOZn up to 0.44 mol L^−1^ ZnO in LO (≈4.5 wt% of ZnO) and a minimal increase in COOZn at concentration above 0.44 mol L^−1^. These results indicate that below 0.44 mol L^−1^ ZnO, part of the acid groups are complexated with zinc ions but there is insufficient ZnO present to neutralise all acid groups. The complete conversion of ZnO below 0.44 mol L^−1^ was confirmed by XRD (see Fig. S5†). Above 0.44 mol L^−1^, an excess ZnO is present and all accessible acid groups are complexated with zinc. Consequently, the tipping point can be used to calculate the concentration of COOH group available for reaction with ZnO: at this point the concentration of COOH is equivalent to twice the concentration of COOZn (and thus ZnO). This approach leads to a calculated [COOH] of 0.88 mol L^−1^ and a molar [COOH]/[COOR] ratio of 0.35. This result means that for roughly every three COOR functionalities, one COOH group is formed. In absence of ester hydrolysis, this corresponds to about one COOH group per triacylglycerol (TAG) unit or LO molecule.

#### Standard addition of sorbic acid

**Fig. 3:**
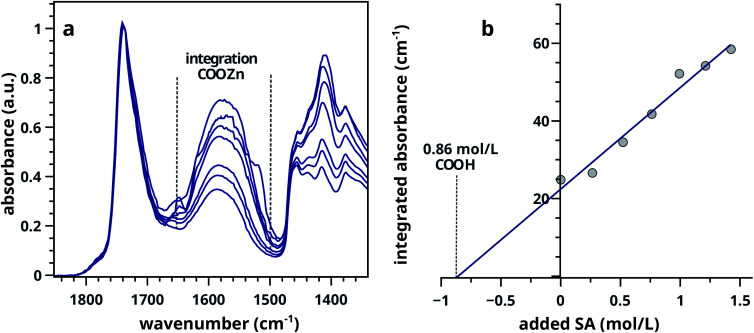
(a) Selection of FTIR spectra of LO–ZnO–SA with added SA from 0.0–1.5 mol L^−1^ of ZnO in LO (0–60 wt%). Dashed lines in indicate band integration limits. (b) Integrated absorption values of COOZn band showing the linear relationship between added SA and COOZn absorption.

To test if additional acid groups in the linseed oil binder would result in the formation of additional COOZn, paints with increasing acidity were synthesised by cross-linking sorbic acid (SA, 2,4-hexadienoate) into ZnO based paint model systems (LO–ZnO–SA). Using the standard addition method, these experiments form a second method of COOH quantification. A constant PVC with a ZnO to LO ratio of 1 : 1 (w/w) was used in this experiment, ensuring an large excess of ZnO. The effect of functionalizing LO with additional acid groups on the COOZn IR absorption band at 1585 cm^−1^ is shown in Fig. 3a. Integrated absorption values of the COOZn band are plotted as a function of added SA in Fig. 3b. Evidently, there is a linear (*R*^2^ = 0.97) relationship between the concentration of acid groups and the COOZn band, confirming the tendency of these systems to form COOZn as long as a source of zinc (ZnO) is present. The concentration of acid groups in pure ZnO–LO (without SA) was calculated to be 0.86 mol L^−1^, resulting in a [COOH]/[COOR] ratio of 0.32 (Fig. 3b). This result is in excellent agreement with our previous result obtained with mixed ZnO/BaSO_4_ paints.

### The origin of COOH formation

Having established a method to determine the COOH concentration in our paint samples, we proceed to investigate whether these acid groups originate from autoxidation of side chain double bonds or from the hydrolysis of ester carbonyl functionalities. Effects of increasing RH and ZnO concentration on COOZn formation are investigated.

#### Autoxidation

The relative amount of autoxidation and the effect of ZnO concentration on COOZn formation was studied. A mixture of alcohols obtained by reducing the esters in linseed oil to alcohols (reduced LO, rLO) was used as a binding medium instead of LO. This approach ensures that all COOZn formation results from autoxidation. Model paint samples containing rLO with varying amounts of ZnO and TiO_2_ fillers (rLO–ZnO–TiO_2_) were made. Coated rutile TiO_2_ fillers were selected for the highly inert properties and low photocatalytic activity.^30^ In order to compare the two mechanisms of COOH formation (Scheme 1), reduced LO containing model paints were compared with samples made with ordinary LO.

**Fig. 4:**
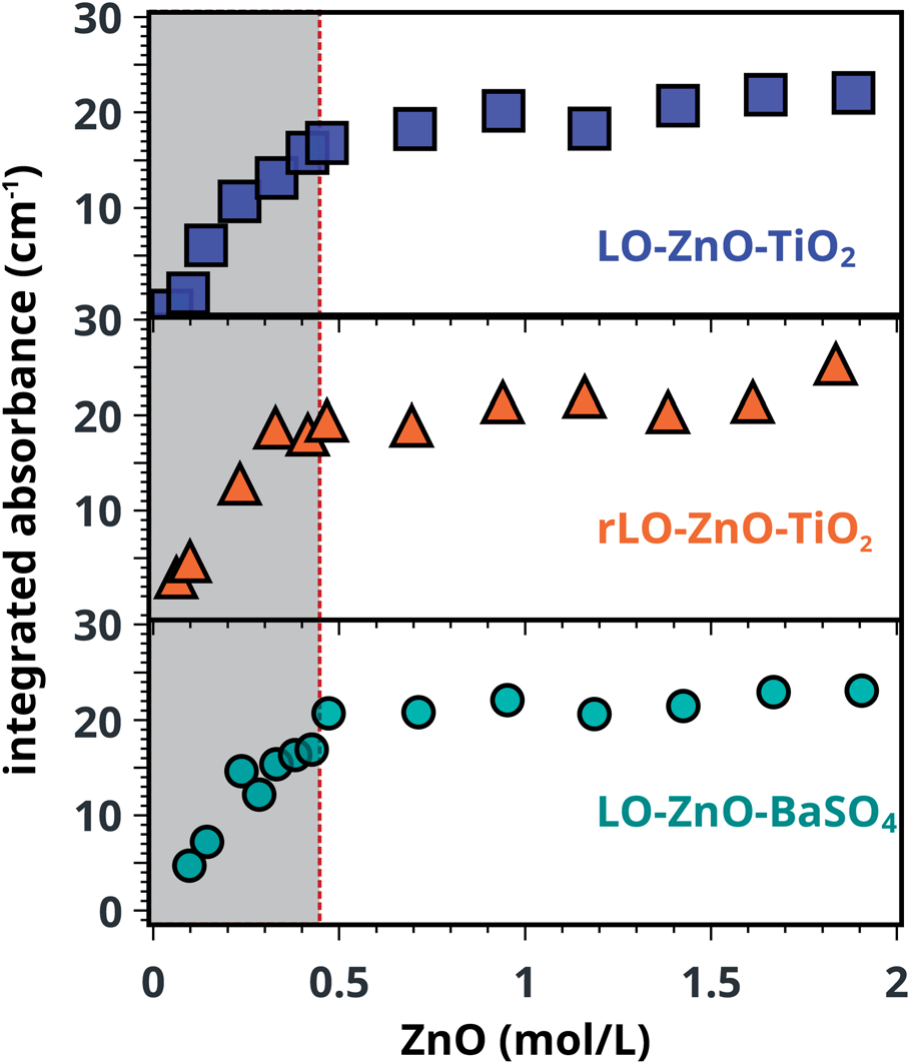
Integrated absorbance of the COOZn band for LO–ZnO–TiO_2_, rLO–ZnO–TiO_2_ and LO–ZnO–BaSO_4_ model systems. The dotted line indicates the previously found tipping point at 0.44 mol L^−1^ ZnO. All IR spectra were normalised on the CH_2_ vibration (2929 cm^−1^) before integration.

Comparing rLO–ZnO–TiO_2_ (triangles) with LO–ZnO–TiO_2_ (squares) in Fig. 4, it is clear that using rLO does not significantly affect COOZn formation at low ZnO concentrations (0.1–2 mol L^−1^). Hence, we conclude that the previously calculated COOH concentration of 0.88 mol L^−1^ is the result of side chain autoxidation (Scheme 1, pathway 2). Fig. 4 also shows that the integrated absorption values of the COOZn band in LO–ZnO–BaSO_4_ (circles) and LO–ZnO–TiO_2_ (squares) are highly similar, verifying the chemical inertness of the chosen TiO_2_ and BaSO_4_ fillers. To confirm that autoxidation is the main pathway leading to the formation of COOH groups in rLO–ZnO–TiO_2_ samples, we cured a mixture of fully saturated triglycerides (tristearin) and ZnO (1 : 1 by wt.) for one week at 60 °C and 12% RH. In this mixture, no COOZn was formed after one week (see Fig. S9†).

#### Hydrolysis

Having established that autoxidation is dominant at low RH, we investigated high RH conditions and increasing ZnO concentrations because these factors likely promote ester hydrolysis. A number of recent publications have already demonstrated the relationship between the presence of ZnO^15^ or high RH conditions and the formation of organic acids in paint extracts.^14,16^ We compared LO–ZnO–TiO_2_ and rLO–ZnO–TiO_2_ paint models cured in dry (12% RH) and wet conditions (77% RH). A large ZnO concentration range of 3–9 mol L^−1^ (30–90 wt% of ZnO in LO) was studied.

**Fig. 5:**
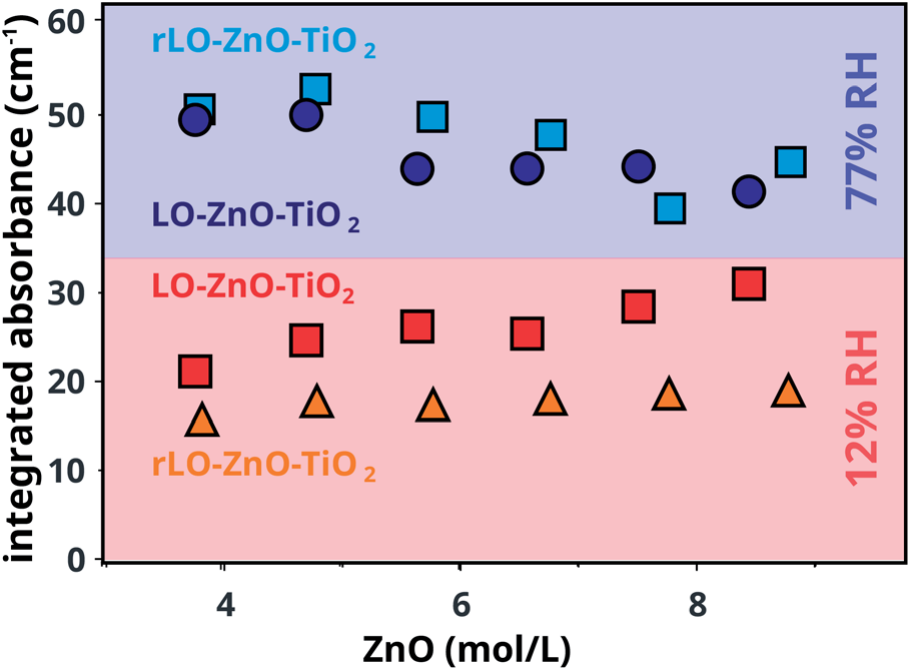
Integrated absorbance of the COOZn band for LO–ZnO–TiO_2_ and rLO–ZnO–TiO_2_ from 30–90 wt% cured in 12% and 77% RH at 60 °C for 7 days. All IR spectra were normalised on the CH_2_ vibration (2929 cm^−1^) before integration.

Integrated absorbance values of the COOZn band at 1585 cm^−1^ for LO–ZnO–TiO_2_ and rLO–ZnO–TiO_2_ cured in dry and wet conditions are plotted as a function of ZnO concentration in Fig. 5. The integrated COOZn absorbance for both systems cured in 77% RH conditions is significantly higher than when cured at 12% RH (Fig. 5). Interestingly, both LO–ZnO–TiO_2_ and rLO–ZnO–TiO_2_ show this effect, indicating that autoxidation is also the dominant pathway for COOH formation at high RH conditions and high ZnO concentrations. Previous research has shown that the oxygen uptake and peroxide decomposition rate in methyl linoleate are, in the first 80 hours, lower at higher water activities, resulting in a lower quantity of carbonyl compounds.^31^ Upon longer autoxidation times of up to two weeks, the production rates of hydroperoxides and conjugated dienoic acids were found to increase with increasing water activities.^32^ Water can thus stabilise and destabilise intermediates during different stages of the autoxidation pathway, resulting in an increased formation of COOH groups for long oxidation times. This can explain the increased COOZn formation at 77% RH conditions in our measurements.

Comparing both systems at 12% RH, a small but significant difference is visible: the integrated absorbance for the COOZn band is consistently larger for LO–ZnO–TiO_2_ and seems to increase with increasing ZnO concentration. It is known that the low concentrations of free FAs (0.5 wt%) can significantly increase the rate of autoxidation in methyl linoleate.^33^ The presence of free FAs in the regular LO may thus explain the formation of more COOZn in LO–ZnO–TiO_2_. This effect is not visible at 77% RH, suggesting that the effects of water on autoxidation are stronger than the effects of free FAs at high humidity. We currently do not have a satisfying explanation for the increase in COOZn with increasing ZnO at 12% RH for LO–ZnO–TiO_2_. Since we have shown that there is no hydrolysis of ester bonds in a mixture of ZnO and tristearin at 12% RH, hydrolysis can not explain this effect.

## Conclusions

The concentration of acid groups in analytically challenging polymerised oil paint models was quantified using ATR-FTIR. Because the formation of COOH groups drives the release of zinc ions from ZnO pigment to form COOZn, this mechanism can be used to determine the concentration of COOH groups available for coordination with zinc ions. The COOH concentration in ZnO paint models was calculated to be roughly one COOH group per TAG unit. This result was confirmed using the standard addition of sorbic acid to linseed oil in excess of ZnO.

At low ZnO concentrations low RH, COOH groups were found to form by autoxidation only. To investigate conditions that likely promote ester hydrolysis, the effects of moisture on COOH formation were studied over a wide range of ZnO concentrations. A strong increase in COOZn was observed at 77% RH compared to 12% RH due to an accelerating effect of water on COOH formation. No hydrolysis was observed under the conditions studied, indicating that oil paints are, in the initial stage of curing, quite resistant to hydrolysis.

Our results demonstrate that smart design of models systems enable a better understanding of oil paint degradation, aiding the improvement of storage and conservation strategies.

## Conflicts of interest

There are no conflicts of interest to declare.

## Supplementary Material

RA-009-C9RA06776K-s001
