# Supplementary material for: The concentration and origins of carboxylic acid groups in oil paint
Source: RSC Adv. 2019 Nov 1;9(61):35559–64. doi: 10.1039/c9ra06776k (PMC9074637; doi:10.1039/c9ra06776k)
Supplement: RA-009-C9RA06776K-s001 [file RA-009-C9RA06776K-s001.pdf]

Electronic Supplementary Material (ESI) for RSC Advances.
This journal is © The Royal Society of Chemistry 2019

The concentration and origins of carboxylic acid groups in oil paint

Lambert Baij,*[a,b] Louise Chassouant,[a] Joen J. Hermans,[a,b] Katrien Keune,[a,b] and Piet Iedema[a]

* *E-mail: C.L.M.Baij@uva.nl*

[a] *Van 't Hoff Institute for Molecular Sciences, University of Amsterdam, PO box 94720, 1090GD Amsterdam, the Netherlands. Tel: +31 (0)20 525 7143*

[b] *Rijksmuseum Amsterdam, Conservation and Science, PO box 74888, 1070DN Amsterdam, the Netherlands.*

List of Figures

S1 IR spectrum polymerised LO 3
S2 Ester band subtraction procedure 4
S3 TiO_2 background estimation and subtraction procedure 5
S4 SEM images of pigment particles 12
S5 $BaSO_4$ normalized XRD spectra for mixed $ZnO/BaSO_4$ paints 13
S6 TiO_2 normalized XRD spectra for mixed ZnO/TiO_2 paints 14
S7 FTIR spectrum of LO and reduced LO 16
S8 FTIR spectrum of polymerised LO and polymerised reduced LO 17
S9 FTIR spectrum of tristearin and tristearin-ZnO 18

List of Tables

1 Composition of LO-ZnO-$BaSO_4$ series 0.5–20% 6
2 Composition of LO-ZnO-TiO_2 series 0.5–20% 7
3 Composition of rLO-ZnO-TiO_2 series 0.5–20% 8
4 Composition of LO-ZnO-SA series 0–60% 9
5 Composition of ZnO-TiO_2-LO series 30–90% 10
6 Composition of rLO-ZnO-TiO_2 series 30–90% 11

FTIR spectrum polymerised LO

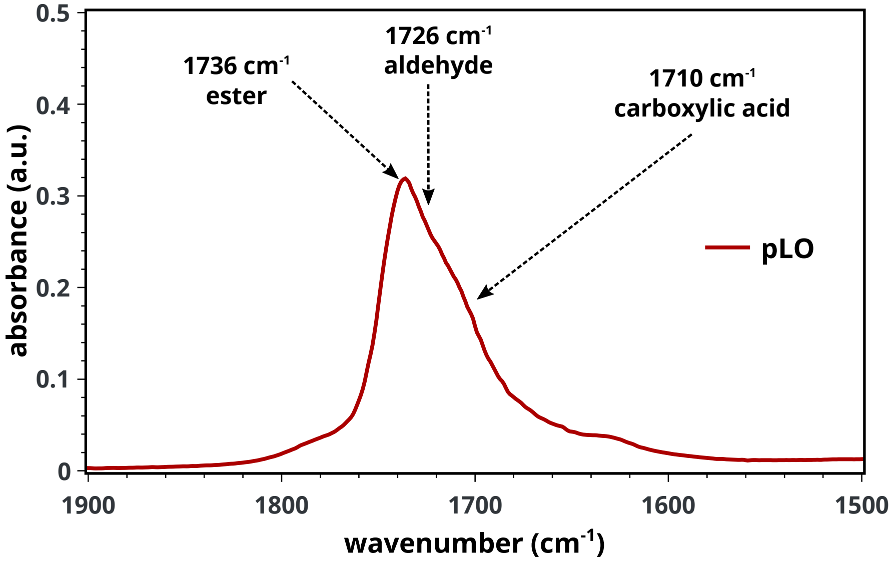

Figure S1 Overlapping IR absorption bands of the asymmetric stretching vibration (ν_a COO) of the ester (COOR, 1738 cm^{-1}), aldehyde (COH, 1726 cm^{-1}) and carboxylic acid (COOH, 1710 cm^{-1}) carbonyl function in polymerised LO.

COOZn IR band integration method

To integrate overlapping absorption bands accurately, data correction and integration algorithms were written in the Wolfram *Mathematica* software, which are available from the authors on request. Since the zinc carboxylate (COOZn) band present in ZnO-LO samples at 1685 cm^{-1} overlaps with the ester carbonyl band (maximum at 1738 cm^{-1}), a band fitting algorithm was applied to subtract the ester band and integrate the COO-Zn band accurately (see Figure S2a). After subtraction of the Pearson type IV band shape[1] fitted to the ester carbonyl band, the area of the COOZn band was calculated (Figure S2b, integration limits are indicated with a dashed line).

Ester band subtraction procedure

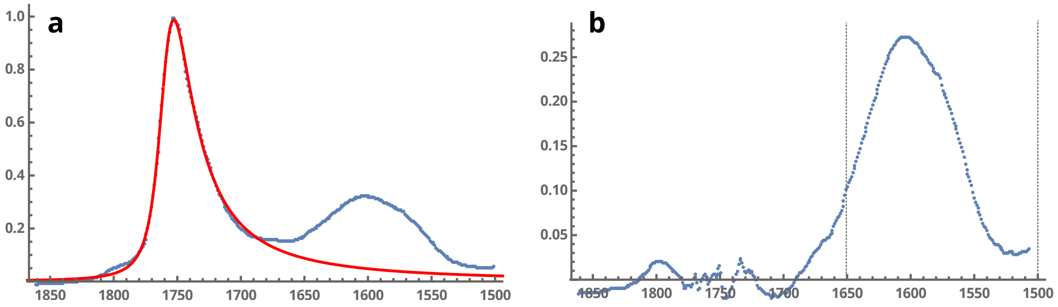

Figure S2 Subtraction procedure for the asymmetric stretching vibration (ν_a CO) of the ester (COOR, 1738 cm^{-1}) in polymerised LO. A Pearson type IV band shape[1] was fitted to the experimental data (a) and subtracted (b) before integration.

TiO_2 background estimation and subtraction procedure

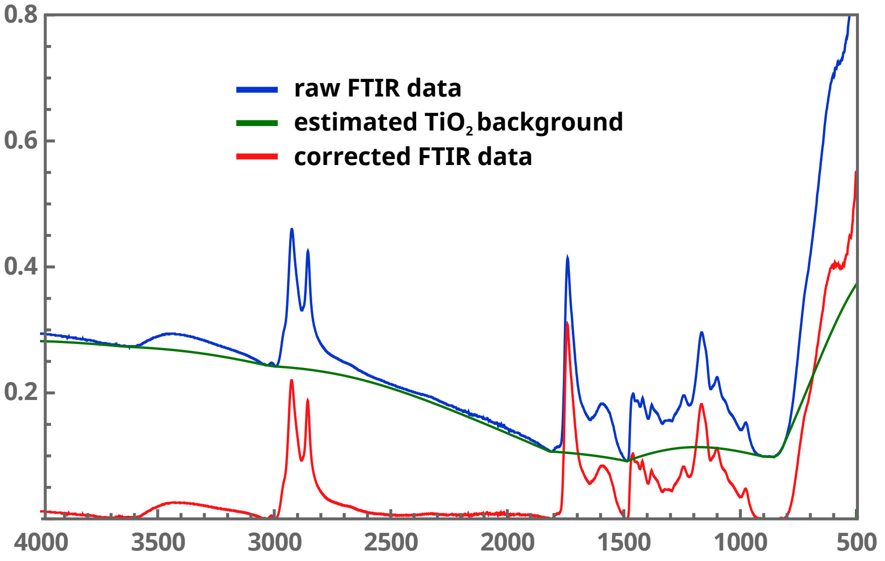

Figure S3 Automated background subtraction procedure using *Mathematica* software. The changing concentration of TiO_2 along the LO-ZnO-TiO_2 samples requires a different background subtraction for every sample. After TiO_2 background estimation, the COO–Zn band area was calculated according to Figure S2.

Composition of LO-ZnO-$BaSO_4$ series 0.5–20%

Sample	Mass LO (mg)	Mass ZnO (mg)	Mass $BaSO_4$ (mg)	[ZnO] mol/L
LO-ZnO-$BaSo_4$-0.5	1000.2	4.80	995.0	0.045
LO-ZnO-$BaSo_4$-1	1000.1	10.2	989.7	0.097
LO-ZnO-$BaSo_4$-1.5	1000.6	15.2	985.2	0.14
LO-ZnO-$BaSo_4$-2.5	1000.3	25.0	975.2	0.24
LO-ZnO-$BaSo_4$-3	1000.3	30.1	970.0	0.28
LO-ZnO-$BaSo_4$-3.5	999.70	34.9	965.1	0.33
LO-ZnO-$BaSo_4$-4	1000.4	40.2	960.1	0.38
LO-ZnO-$BaSo_4$-4.5	999.50	44.9	955.0	0.43
LO-ZnO-$BaSo_4$-5	999.30	49.8	950.0	0.47
LO-ZnO-$BaSo_4$-7.5	999.80	75.1	924.9	0.71
LO-ZnO-$BaSo_4$-10	1000.5	100.3	900.4	0.95
LO-ZnO-$BaSo_4$-12.5	999.70	124.8	874.8	1.2
LO-ZnO-$BaSo_4$-15	1000.2	149.9	850.4	1.4
LO-ZnO-$BaSo_4$-17.5	999.80	175.2	824.9	1.7
LO-ZnO-$BaSo_4$-20	1000.2	200.0	799.7	1.9

Table 1 Composition of LO-ZnO-$BaSO_4$ series 0.5–20% with a constant (1:1) pigment to oil ratio by weight and a PVC of 17–18%.

Composition of LO-ZnO-TiO_2 series 0.5–20%

Name	Mass LO (g)	Mass ZnO (g)	Mass TiO_2 (g)	[ZnO] mol/L
LO-ZnO-TiO_2-0.5	999.8	5.2	994.7	0.049
LO-ZnO-TiO_2-1	1000.3	9.9	990.1	0.092
LO-ZnO-TiO_2-1.5	1000.3	15.1	985.0	0.14
LO-ZnO-TiO_2-2.5	1033.2	25.0	975.1	0.23
LO-ZnO-TiO_2-3.5	1033.2	35.3	965.0	0.33
LO-ZnO-TiO_2-4.5	1033.2	44.2	955.0	0.41
LO-ZnO-TiO_2-5	999.6	49.8	950.3	0.47
LO-ZnO-TiO_2-7.5	999.4	75.0	925.2	0.70
LO-ZnO-TiO_2-10	1000.3	100.3	900.0	0.94
LO-ZnO-TiO_2-12.5	1000.2	124.8	874.9	1.2
LO-ZnO-TiO_2-15	999.9	149.9	850.0	1.4
LO-ZnO-TiO_2-17.5	1000.6	174.9	825.1	1.6
LO-ZnO-TiO_2-20	1000.1	200.3	800.1	1.9

Table 2 Composition of LO-ZnO-TiO_2 series 0.5–20% with a with a constant (1:1) pigment to oil ratio by weight and a PVC of of 17–18%.

Composition of rLO-ZnO-TiO_2 series 0.5–20%

Name	Mass LO (mg)	Mass ZnO (mg)	Mass TiO_2 (mg)	[ZnO] mol/L
rLO-ZnO-TiO_2-0.5	199.5	1.3	198.4	0.059
rLO-ZnO-TiO_2-1	200.4	2.1	197.8	0.10
rLO-ZnO-TiO_2-1.5	666.4	10.0	646.3	0.14
rLO-ZnO-TiO_2-2.5	500.2	12.4	483.8	0.23
rLO-ZnO-TiO_2-3.5	500.1	17.5	481.7	0.33
rLO-ZnO-TiO_2-4.5	333.4	14.8	321.7	0.41
rLO-ZnO-TiO_2-5	332.8	16.6	321.5	0.47
rLO-ZnO-TiO_2-7.5	333.3	24.8	319.6	0.69
rLO-ZnO-TiO_2-10	251.4	25.4	239.8	0.94
rLO-ZnO-TiO_2-12.5	250.2	31.3	238.4	1.2
rLO-ZnO-TiO_2-15	200.7	30.1	191.0	1.4
rLO-ZnO-TiO_2-17.5	200.1	35.0	190.2	1.6
rLO-ZnO-TiO_2-20	200.7	40.1	189.4	1.8

Table 3 Composition of LO-ZnO-TiO_2 series 0.5–20% with a PVC of of 17–19%

Composition of LO-ZnO-SoA series 0–60%

Name	Mass LO (mg)	Mass ZnO (mg)	Mass SoA (mg)	[SoA] mol/L
LO-ZnO-SA-0	1033.2	1006.1	0	0
LO-ZnO-SA-10	1004.1	998.3	38.3	0.26
LO-ZnO-SA-20	1000.7	999.4	77.1	0.52
LO-ZnO-SA-30	999.4	1000.0	115.2	0.76
LO-ZnO-SA-40	1000.5	1000.2	153.8	0.99
LO-ZnO-SA-50	1000.6	1000.7	192.1	1.2
LO-ZnO-SA-60	999.7	1000.5	230.9	1.4

Table 4 Composition of sorbic acid (LO-ZnO-SA) series 0–60% with a constant (1:1) pigment to oil ratio by weight.

Composition of ZnO-TiO_2-LO series 30–90%

Name	Mass LO (mg)	Mass ZnO (mg)	Mass TiO_2 (mg)	[ZnO] mol/L
LO-ZnO-TiO_2-30	1000.4	302.1	753.7	2.83
LO-ZnO-TiO_2-40	1000.2	401.3	682.0	3.76
LO-ZnO-TiO_2-50	1002.2	501.1	605.0	4.69
LO-ZnO-TiO_2-60	998.90	601.1	530.9	5.64
LO-ZnO-TiO_2-70	1000.6	703.1	457.9	6.59
LO-ZnO-TiO_2-80	1005.1	800.1	381.0	7.47
LO-ZnO-TiO_2-90	1001.1	902.2	308.0	8.45

Table 5 Composition of ZnO-TiO_2-LO series 30–90% with a constant PVC of 18%.

Composition of rLO-ZnO-TiO_2 series 30–90%

Name	Mass LO (mg)	Mass ZnO (mg)	Mass TiO_2 (mg)	[ZnO] mol/L
rLO-ZnO-TiO_2-30	201.3	59.9	139.9	0.140829
rLO-ZnO-TiO_2-40	201.2	78.7	120.2	0.231425
rLO-ZnO-TiO_2-50	201.4	100.3	101.5	0.330388
rLO-ZnO-TiO_2-60	201.0	120.1	81.0	0.413875
rLO-ZnO-TiO_2-70	201.5	139.7	59.5	0.466398
rLO-ZnO-TiO_2-80	199.7	159.0	40.3	0.703532
rLO-ZnO-TiO_2-90	201.5	180.5	21.1	0.941005

Table 6 Composition of rLO-ZnO-TiO_2 series 30–90%. Pigment volume concentration 14–18%.

SEM images of pigment particles

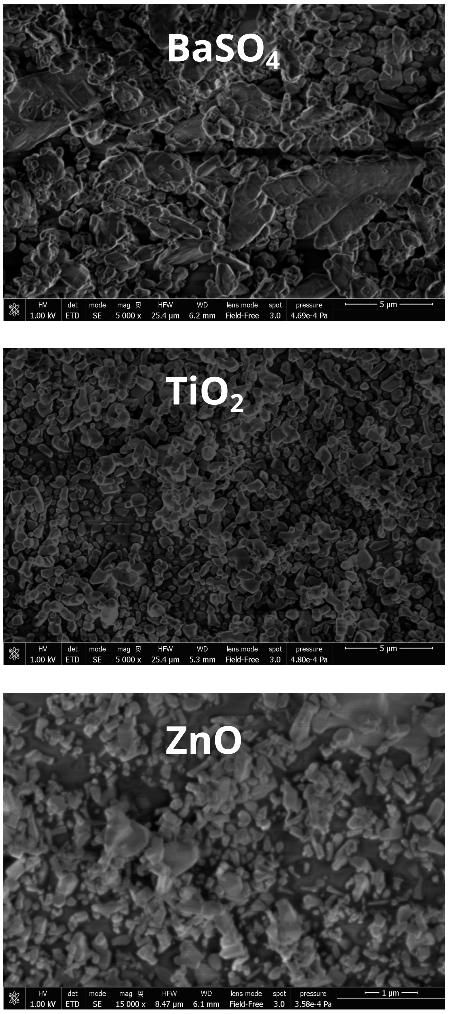

Figure S4 SEM images of $BaSO_4$, TiO_2 and ZnO pigments. $BaSO_4$ shows a more heterogeneous size distribution compared to TiO_2 but in the same $1--5$ μm range, ZnO is smaller (≈ 0.5 μm).

$BaSO_4$ normalised XRD spectra for ZnO/$BaSO_4$ paints

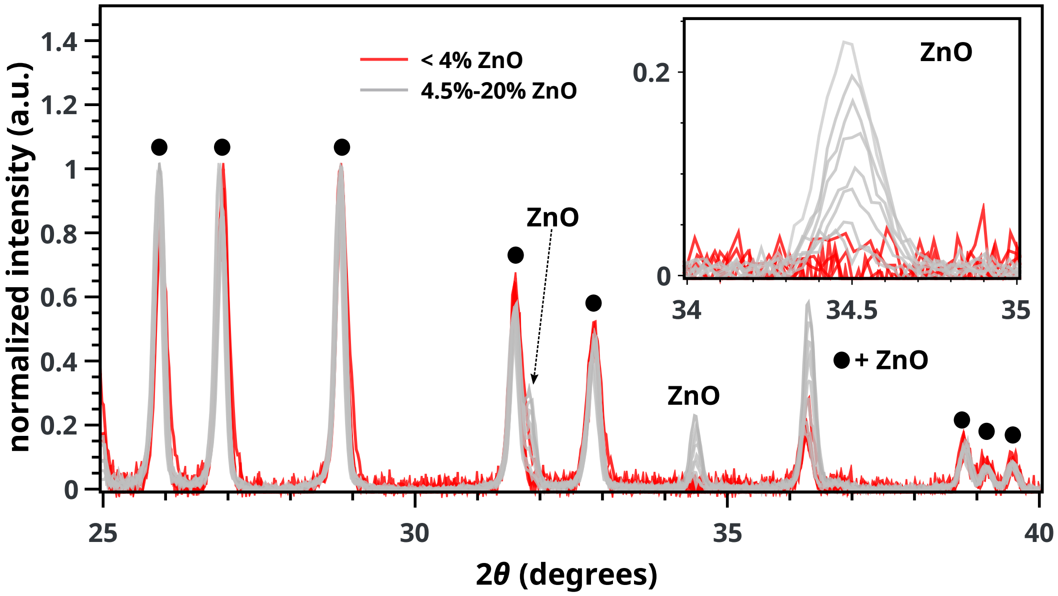

Figure S5 $BaSO_4$ normalised XRD spectra for mixed ZnO/$BaSO_4$ paints containing 0.5–20 wt % of ZnO. Inset shows that ZnO is completely consumed below $\approx$ 4.5 wt%. $BaSO_4$ peaks are marked with •.

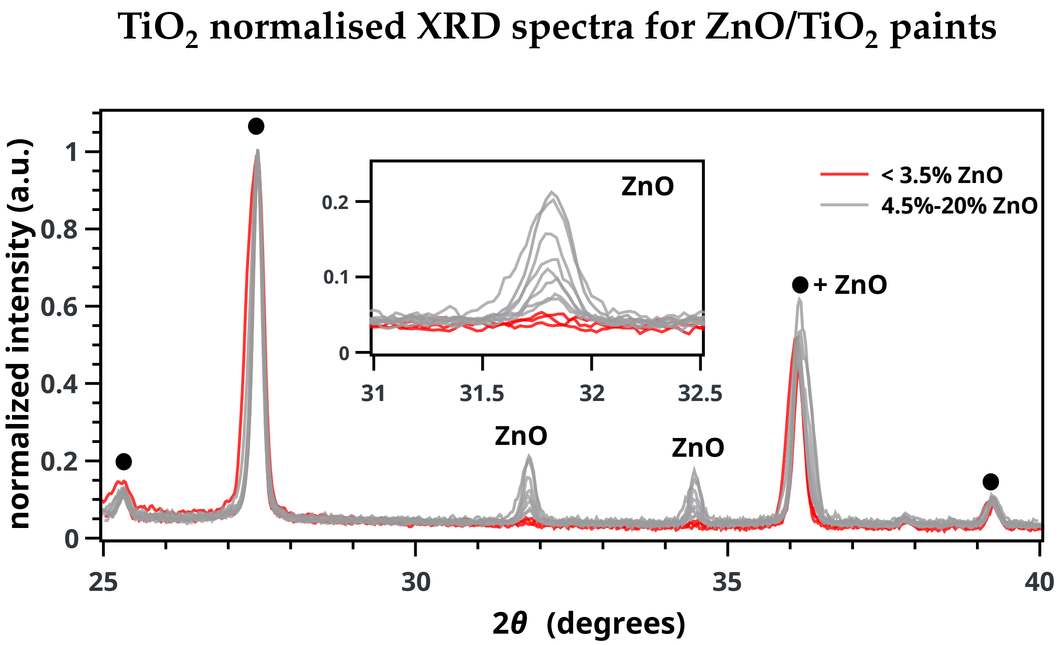

Figure S6 TiO_2 normalised XRD spectra for mixed ZnO/TiO_2 paints containing 0.5–20 wt % of ZnO. Inset shows that ZnO is completely consumed below $\approx$ 4.5 wt%. TiO_2 peaks are marked with •.

Reduction of linseed oil with $LiAlH_4$

The reduction of linseed oil with lithium aluminium hydride $LiAlH_4$ was done according to slightly modified literature procedures.[2,3] $LiAlH_4$ (5.2 g, 138 mmol) was dispersed in 225 ml cold (0 °C) tetrahydrofuran (THF) under nitrogen atmosphere. Cold-pressed untreated linseed oil (LO, Kremer Pigmente, 15 g, 17 mmol) was added while stirring the reaction in a ice bath. The reaction mixture was then stirred continuously at allowed to reach room temperature. After 17 h, the mixture was cooled again to 0 °C and 40 ml of 2-propanol, ethanol and 20 ml of water was *slowly* added. The mixture was stirred for 15 min and filtered using a Büchner funnel. The solvent was removed under reduced pressure, dissolved in dichloromethane and filtered over $MgSO_4$. The solvent was removed under reduced pressure to give the mixture of alcohols (14 g, 93% yield). ^1H NMR (300 MHz, $CDCl_3$) δ 5.50 − 5.21 (m, -CH=CH-), 3.64 (t, $J = 6.6$ Hz, $-CH_2OH$), 2.81 (t, J = 5.8 Hz, $-CH{=}CHCH_2CH{=}CH-$), 2.17 − 1.96 (m, $CH{=}CHCH_2C^-$), 1.56 (s, $-CH_2CH_2OH$), 1.37 − 1.28 (br, $-CCH_2C-$), 0.97 (t, $J = 7.5$ Hz, CH_3).

FTIR spectrum of LO and reduced LO

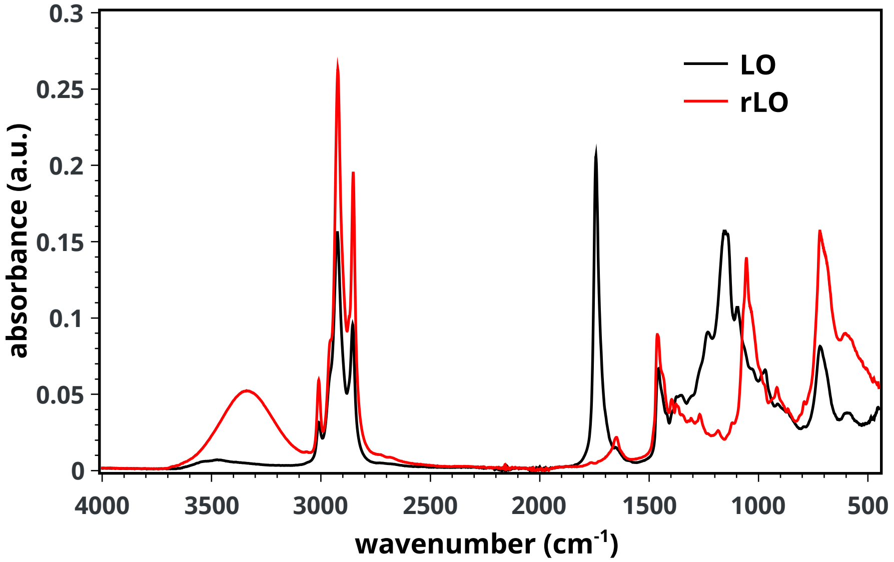

Figure S7 FTIR spectrum of LO and reduced LO (**rLO**), clearly showing the reduction of all esters (1736 cm^{-1}) for **rLO** and the presence of the OH stretch (3400 cm^{-1}, broad) in the mixture of alcohols.

FTIR spectrum of polymerised LO and polymerised reduced LO

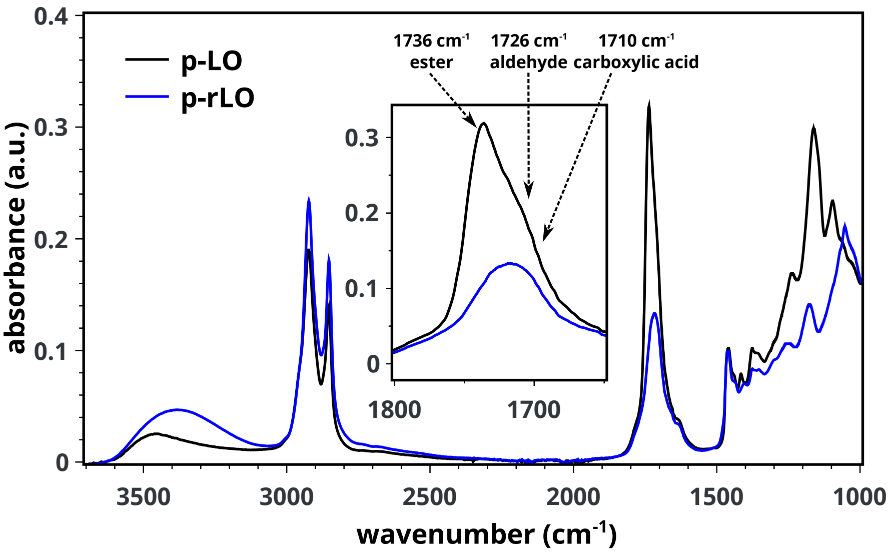

Figure S8 FTIR spectrum of polymerised LO (**p-LO**) and polymerised reduced LO (**p-rLO**).

FTIR spectrum of tristearin and tristearin-ZnO

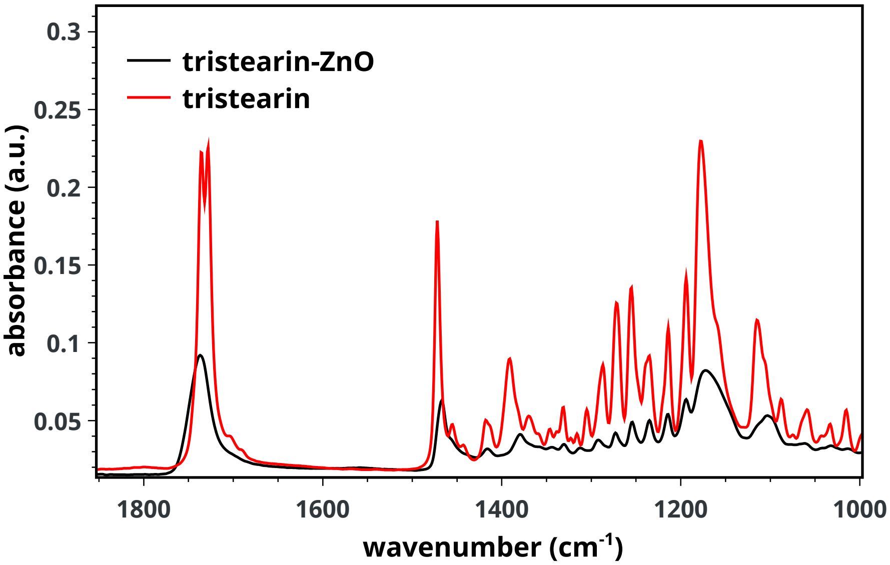

Figure S9 FTIR spectrum of tristearin and tristearin-ZnO, clearly showing that 1 week at 60°C and 12% RH did not lead to the formation of significant amounts of COOZn. A split ester carbonyl band is observed for tristearin due to the presence of different polymorphs.[4,5]

References

[1] H. Keles, A. Naylor, F. Clegg and C. Sammon, *The Analyst*, 2014, **139**, 2355–2369.

[2] H. M. Walborsky and C. Colombini, *The Journal of Organic Chemistry*, 1962, **27**, 2387–2390.

[3] T. Tsujimoto, H. Uyama and S. Kobayashi, *Macromolecules*, 2004, **37**, 1777–1782.

[4] J. Yano and K. Sato, *Food Research International*, 1999, **32**, 249–259.

[5] E. Da Silva, S. Bresson and D. Rousseau, *Chemistry and Physics of Lipids*, 2009, **157**, 113–119.
